# Urban environments’ impacts on aging in post-industrial cities: reviewing the pathways to healthy aging

**DOI:** 10.3389/fmed.2025.1613587

**Published:** 2025-09-10

**Authors:** Hongsheng Zhao, Marco Spada, Ilaria Bortone

**Affiliations:** 1 School of Technology, Business, and Arts, University of Suffolk, Ipswich, United Kingdom; 2 School of Computing, Engineering and Physical Sciences, University of the West of Scotland, Paisley, United Kingdom; 3 APS Public Health Environment and Social Equity - PLANET, Bari, Italy

**Keywords:** healthy ageing, urban environment, age-friendly cities, and communities, active ageing, post-industrialization

## Abstract

Post-industrial cities are characterized by an aging population, deteriorating urban infrastructure, and a range of socio-environmental challenges, including pollution, economic decline, and out-migration. These conditions, alongside the legacies of industrial decline, make aging in such environments considered particularly challenging when studied through the lens of conventional analytical frameworks such as active aging and age-friendly cities and communities (AFCC). By examining the literatures critically and analyzing unique spatial and social features of the environment of post-industrial cities, the paper expands the discussion regarding the impacts of environments on the healthy aging process within a post-industrial context. Based on 42 selected articles as evidences in exploring association of healthy aging and post-industrial built environment, this review implies that despite the negative perceptions, post-industrial city and community’s strong social networks within their long-established communities identities hold untapped potential to play positive roles in the pathways to healthy aging. This fresh perspective challenge prevailing assumptions on aging in post-industrial cities, offering a more nuanced understanding of the strengths and opportunities inherent in post-industrial contexts for supporting the healthy aging of elderly residents.

## 1 Introduction

Deindustrialization and aging are two major trends in the developed world in 21st century (1). The population aged 65 and over is anticipated to reach 30% by 2026 in Europe [(2), p. 18], with an increasing number of aged people living in post-industrial context, as many countries entered post-industrial societies (3). In other significant developing economics such as China, the population has started to decline in 2022 (4), making the country an earlier entry than the predicted 2040 into an “aged society”– defined as a country where the percentage of people aged 65 or older is between 15% and 20% of the total population (5).

Meanwhile, at a local level, post-industrial cities are characterized by socio-environmental challenges such as pollution, declining infrastructure, and reduced access to services, all of which exacerbate health inequalities, particularly compared to rapidly growing urban centers (6, 7). Although extensive research exists on environmental health impacts, limited attention is paid to contexts like post-industrial cities or Global South regions, highlighting the need for a geographically diverse analysis. Moreover, studying the mechanisms of healthy aging in post-industrial cities has implications for studying healthy aging in more diversified surroundings (8), for example, healthy aging in rural communities and healthy aging in left-behind places.

By asking, “How does the urban environment impact the healthy aging process in post-industrial cities?” This paper aims to examine the mechanisms and dimensions influencing healthy aging in post-industrial realities, addressing the existing gaps of the limited exploration of socio-environmental interactions and their implications on aging populations in a post-industrial context.

The paper is organized as follows: We first lay out a knowledge foundation, including the existing framework for healthy aging, the features of the aging experience in post-industrial cities, and an explanation of the literature sampling methods. In the Section “4 Analysis,” the literature identified is further organized into three themes, essentially connecting the key aspects of “post-industrial cities,” “healthy aging” and “urban environment,” capturing the key findings of the analysis while connecting the analysis of built and socio-economic environments under post-industrial realities. The study is concluded by identifying the potential of the post-industrial environment with policy recommendations and proposals for future research directions.

## 2 Background

### 2.1 Frameworks for healthy Aging

Aging is a ubiquitous though dissimilar. Experiences for communities in different urban settings (1). Existing analytical framework in the past two decades from gerontology – the scientific study of aging [(9), p. 2], has taken on various lenses in analyzing the aging process as well as the outcomes: Compression of morbidity and mortality theories highlights the outcome (10) while life-course perspective on elderly lives (11) emphasized on the aging process; social determinant of health framework (12) pinpointed the importance of cultural and social factors while biopsychosocial model of aging (13) added on the biological and psychological parameters.

Some frameworks takes on a broader scope such as the political economy of aging (14), whilst theories like successful aging framework (15) adopts a microscope that is embedded in individual’s aging experience.

Apart from the well-established analytical frameworks aforementioned, there is a new trend of critical gerontology (16) that questions societal norms (17), power structures (18), and inequalities that influence how we view and treat older adults by engaging feminist (19), race, and critical social theories.

As post-industrial communities are often exclusively situated in urban settings (20), two mainstream frameworks for comprehensively understanding aging are particularly relevant in highlighting the urban environment’s dynamics: active aging and aging-friendly cities and communities (AFCC). The review article will present and assess these two frameworks before focusing on empirical case studies from the environment, planning, and health literature.

#### 2.1.1 Active aging

Active aging refers to “the process of optimizing opportunities for health, participation and security in order to enhance quality of life as people age” [(21), p. 12]. Deployed by World Health Organization (21) and the European Commission (22), the theory of active aging is inspired by Havighust’s Activity Theories on Aging (23), challenging and complementing Disengagement Theories on aging, where the aging process was previously understood as the disengagement of interaction between the aging person and a wider social network in the social system (24, 25).

Under active aging framework, physical environments are considered one of the six major determinants for active aging, particularly in achieving the UN healthy aging principles of “independence, participation, dignity, care and self-fulfilment” (26).

**FIGURE 1:**
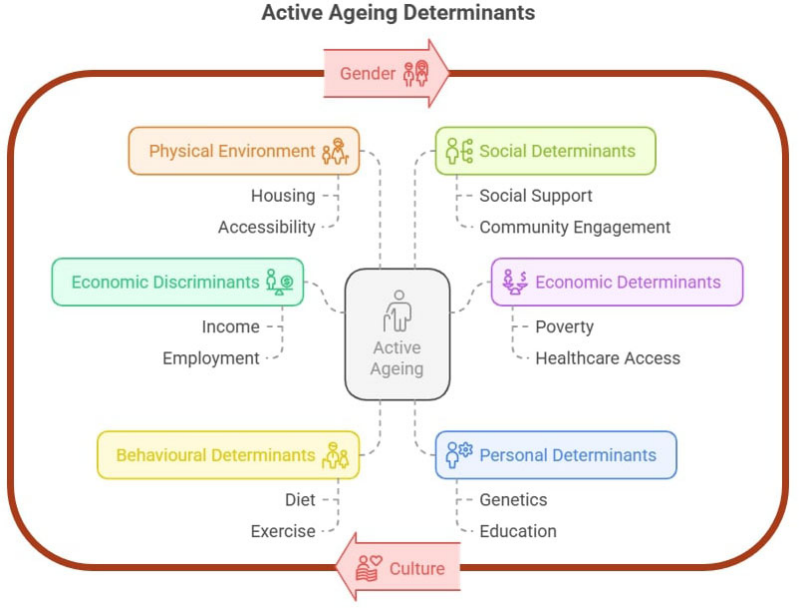
The determinants of active ageing framework.

In Figure 1, physical environment as a determinant of active aging is closely related to other determinants while entangled with the overarching themes of culture and gender. To illustrate with an example, relatively more straightforward access to green spaces can foster healthier lifestyles, including regular physical exercise – as personal determinants, while abundant public spaces and pedestrian-friendly streets can cultivate meaningful personal interaction with neighbors – as social determinants. In contrast, living in an unsafe environment with mobility barriers inevitably further isolates the elderly, increases mobility problems, and reduces the possibility of getting social support while aggravating the possibility of negative mental issues such as depression. There are also peculiar factors when considering built environments for the aged group, as they are more prone to fall – an increased cause of injuries, immobility and health (27).

The Active Aging framework provides an overarching paradigm for analyzing aging under spatial and non-spatial factors, highlighting both opportunities and challenges. The framework takes the interwoven features of various determinants into account and informs researchers to explore the intrinsic connections. It also serves as theoretical foundations for various empirical research.

#### 2.1.2 Aging-friendly cities and communities (AFCC)

The aging process is increasingly contextualized in the scales of cities and communities. An effective way to deliver the outcome of healthy aging is to engage cities and communities to become more age friendly. The AFCC framework developed by the World Health Organization (WHO) has an emphasis on spatial dimensions covering various scales, including the urban environment that spans domestic and public places where the elderly individual has interactions, as shown in Table 1, with spatial-related sub-categories marked by an asterisk (*):

**TABLE 1:** WHO’s checklist for building age-friendly cities.

Checklist criterion	
Outdoor spaces and buildings*	Social participation*
Environment*	Accessibility of events and activities
Green space and walkways*	Affordability
Outdoor seating*	Range of events and activities
Pavements*	Facilities and settings*
Roads*	Promotion and awareness of activities
Traffic*	Addressing isolation*
Cycle paths*	Fostering community integration
Safety*	**Respect and social inclusion**
Service*	Respectful and inclusive services
Buildings*	Public images of aging
Public toilets*	Intergenerational and family interactions
Transportation*	Public education
Affordability	Community inclusion
Reliability and frequency	Economic inclusion
Travel destinations*	**Civic participation and employment**
Age-friendly vehicles	Volunteering options
Specialized services	Employment options
Priority seating	Training
Transport drivers	Accessibility
Safety and comfort	Civic participation
Transport stops and stations*	Valued contributions
Information	Entrepreneurship
Community transport	Pay
Taxis	**Communication and information**
Roads*	Information offer
Driving competence	Oral communication
Parking*	Printed information
Housing*	Plain language
Affordability	Automated communication and equipment
Essential services	Computer and the Internet
Design*	Community support and health services*
Modifications*	Service accessibility*
Maintenance*	Offer of services
Aging in place	Voluntary support
Community integration	Emergency planning and care
Housing options*	
Living environment*	

*Category and sub-category directly associated to spatial element Source: WHO (27). Retrieved from: The WHO Age-friendly Cities Framework - Age-Friendly World.

As shown in Table 1, the AFCC frameworks focused on the experience of “aging in place” (28), taking globalization’ impacts on contrasting urban settings under different geographical and regional development conditions. Moreover, the paralleled sub-categories of AFCC are not mutually exclusive; in fact, they may overlap on certain points. Furthermore, the framework is a place-based model as it emphasizes spatial features and is embedded into the spaces of local communities.

The AFCC framework highlights the importance of empowering elderly individuals to shape the built environment and their communities actively, positioning them as potential agents of change when supported by adequate urban structures and local services (29). The accompanying checklist serves as a practical guide for policymakers, facilitating the integration of age-friendly principles into town planning and urban design. However, the framework has faced criticism regarding the representativeness of its categories, given its evidence derived from thematic analyses of 30 globally significant cities, most of which are located in the Global North. Smaller or less prominent cities may face unique challenges not adequately addressed within this framework.

While both frameworks have confirmed the importance of urban environments’ significance in delivering healthy aging outcomes, the following section examine the peculiar characteristics of aging process in the post-industrial cities and communities.

### 2.2 Aging in the post-industrial environment

#### 2.2.1 Industrial cities and post-industrial cities

What exactly are “post-industrial cities,” and what are their features compared to the industrial cities that preceded them? While an industrial city is defined as “a city in which the municipal economy is centered around industry, with significant factories or production facilities, at least historically,” the boundaries distinguishing post-industrial cities from industrial ones remain ambiguous (6).

For the term “post-industrial cities,” “industry” refers explicitly to the secondary sector in the Three-Sector Model of economics, which involves manufacturing raw materials into goods. This production process is further categorized into heavy and light industries (30). Post-industrial sites can be classified based on the types of previous industries of the city, such as steel mills, mining sites, manufacturing factories, or large construction sites, which are typically associated with heavy industry. Post-industrial cities may also have a dominant historical industry (e.g., Taranto for steel production, Rybnik for coal mining, or Dongguan for manufacturing). Former industrial activities, especially those involving chemical or biological processing, often cause a legacy of environmental pollution, reinforcing the stereotypical image of the post-industrial city.

#### 2.2.2 The transformative process of deindustrialization

Historically, industrial cities’ urban development has not prioritized the needs of an aging population (31). The labor force attracted to industrializing cities by the agglomeration effects has typically been young, single, profit-driven, and industrious (32). This demographic advantage of burgeoning industrial cities shifted with the onset of deindustrialization, a process often originating as an economic phenomenon at regional or state levels and intensifying its intrinsic impacts on specific industrial cities.

Deindustrialization refers to the social and economic changes caused by reducing or removing industrial capacity or activity in a country, region, or city (33), particularly in heavy industry or manufacturing (34, 35). Post-industrial societies and communities are the outcomes of deindustrialization on varying scales. According to Bell (3), a post-industrial society emerges when the service sector generates more wealth than the manufacturing sector. Whilst post-industrial cities and communities can be of varied spatial morphology and socio-economic conditions, in this review article, we define post-industrial communities as smaller clusters of people and spaces within deindustrialized cities or regions.

#### 2.2.3 Features of the post-industrial cities

Post-industrial cities and communities are often portrayed as left-behind places (34), or as Kahn depicted, as “dangerous, dirty, poo, and corrupt shell of their former industrial glory”(2021, p. 9), these perceptions can be raised from specific neighborhood where there are reduced job opportunities (36), high unemployment rate and social unrest (37), out-migration of the younger, skilled labor (38), contaminated and toxic environment (39), decaying and dilapidated urban environment (40). The decline of post-industrial communities often contrasts with the climax of its heyday when the local industrial sector flourished. Typical post-industrial regions and nations are the UK, Western Europe, and the USA (41), though post-industrial communities are also prevalent in developing regions and countries.

From the built environment perspective, the post-industrial realities are manifested into multiple dimensions: post-industrial cities often have an environment under constant transformation, as the establishment of major industrial infrastructure has often left recognizable marks on the cityscape, land use patterns, and urban density (31). Regarding urban morphology, post-industrial cities have a distinctive mix of avant-garde and heritage architectures.

From a socio-economic perspective, post-industrial areas are typically unproportionally impacted by austerity economic policies of the states (42). Under the austerity urbanism (43), groups framed as economically unproductive may lead to displacement and denial of access to the state welfare system, including social housing. Regeneration initiatives lead by private sectors turned to favor those who can afford, such as university students and young professionals, further marginalizing the socio-economically vulnerable groups such as the elderly into enclaves with unfavorable living conditions (43).

#### 2.2.4 Health impacts of built environment on the elderly

The elderly population of post-industrial cities and communities are impacted by spatial and socio-economic characteristics (44), with both aspects of characteristics interwoven. The term built environment refers to the human-made surroundings where people live, work, learn, and play, encompassing the scales of design and layout of the cities to the constructive features of public space and urban infrastructures such as transportation (45, 46). The contrast term is the natural environment or “natural nature,” emphasizing the environment’s non-human dimension (31), while when referring to environment in the cities, it is usually considered a combination of both spatial and socio-cultural elements (47).

Built environments have impacts on both the physical and mental health of residents through direct or indirect mechanisms. On the one hand, the quality of the designed, built environment significantly impacts physical health. For example, public spaces with accessible features and facilities can facilitate physical activities, preventing injuries caused by falling – which is one of the highest external factors for mobility for the elderly population (10). Effective transportation system provides in-time access to health care and emergency services (48), thus directly contributing to the physical health conditions of the aged population. Moreover, a built environment could facilitate social interactions in public spaces (49), fostering community engagement, which testified to healthy mental functions of the elderly (50) and reduces loneliness (51). On the contrary, a hostile built environment with pollution, unsafe places, and the lack of facilities of functional and aesthetic features will discourage physical presence and social connections building, thus unproductive to the physical and mental well-being of the elderly (45, 52).

#### 2.2.5 Aging in place in post-industrial cities

“Aging in place” refers to the elderly experiencing the aging process in their household, usually living independently with or without the support and shared living with their families (53). The opposite of “aging in place” is usually referred to as “relocation for care,” or “moving for institutional care” (28). While studies indicate a general preference for aging in place over institutional care, this preference may often be a result of limited mobility or lack of support services. Urban policies should prioritize accessible housing and localized care to address these constraints. Studies revealed that the elderly have an overall positive perception of aging in place, in contrast to assisted living, nursing homes, retirement communities or moving-in with families (54). It is essential to identify and differentiate the reasons that lead to aging in place: it can be either an independent choice by the elderly or due to a lack of mobility and assisted services, leaving people behind.

Due to the lack of mobility caused by the features of post-industrial cities (55), aging in these cities is more likely to be aging in place if left behind, which means the most significant proportion of the aging process happened locally in the neighborhood, implying an acute demand for local services and facilities. Furthermore, aging in place exposes the elderly more to the local neighborhood-built environment, thus potentially amplifying the importance of the local built environment’s health impacts on those groups experiencing aging-in-place compared to their counterparts with higher inter-region mobility. Moreover, as evidenced by migration studies of the elderly, the socio-economically disadvantaged groups of senior citizens are more likely to be left behind (56). Elderly who reported an inability to adapt to city lifestyle are also more likely to stay behind (57, 58), gender and ethnic features also impact the aged individual’s migratory decision and settlement intention (59, 60). This aging-in-place phenomenon in post-industrial communities has indicated a widening gap in understanding the intrinsic impacts of the environment on the aging process, which are detrimental to the increasing population’s welfare. The following section will present the sampling method of this study.

## 3 Sampling method

The review strategically clusters literature into three areas: (1) reviews of previous literature reviews, (2) reviews of evidence identifying associations between typical environmental factors and healthy aging, and (3) analyses of the social characteristics of post-industrial communities and their impacts on aging in such spaces –to comprehensively address the diverse impacts of urban environments on aging in post-industrial contexts.

This review employs a two-phase sampling process using the Journal Citation Reports© and related indices. This strategy ensures a focus on high-impact, peer-reviewed studies, though including non-Western databases could enhance the representativeness of findings.

In the first phase, a list of journals was compiled from four major social science and humanities indices: the Science Citation Index (SCI), the Social Science Citation Index (SSCI), the Science Citation Index Expanded (SCIE), and the Emerging Sources Citation Index (ESCI). This step aimed to identify journals relevant to the research scope. A quality assessment was then conducted, evaluating the selected journals’ credentials, methods, and citation metrics.

In the second phase, the search was conducted using strategically identified keywords, refined through three sources: data from the identified journals in Clarivate, Google Scholar, and the Taylor & Francis Journal Article Search Browser. Google Scholar was chosen because it is the largest cross-institutional, free journal database with many open-access results. Additionally, most journals identified in phase one that align with the research scope are published by Taylor & Francis, justifying the searches on the Taylor and Frances Repository.

The list of keywords used in the search process is provided in Table 2.

**TABLE 2:** Literature search strategies.

Source	Article type	Keywords
Clarivate Indexed Journals	Review article Empirical article	“Post-industrial” OR “post-industrial cities” OR “deindustrialization” Environment Health Ag(e)ing
Google Scholar	Review article Empirical article	“Post-industrial” OR “post-industrial cities” Environment OR “urban environment” Health OR healthy ag(e)ing
Tylor & Francis Articles Repository	Empirical article	Environment OR “built environment” OR “social environment” Healthy ag(e)ing

Source: authors-own.

This review prioritizes English-language, peer-reviewed journals for consistency and quality assurance while acknowledging the potential exclusion of valuable insights from non-English or regional studies: only journals published in English are selected for this review. The review includes journal articles published from January 1, 2000, onward exclusively, provided they underwent a double-blind peer review process. Unpublished working papers, conference proceedings, and commentary articles were excluded from this review. These criteria are imposed to ensure the credibility of the data sources.

In Table 3 the primary focus was on journals related to the built environment, whilst genealogy-focused journals with specific relevance to the built environment were also solicited while progressing into the literature search. The rationale for this approach is that if a built environment journal addresses health-related topics, the evidence will align with the sampling framework of this review. Conversely, genealogy-focused articles do not necessarily include elements of the built environment, which justifies separating these during the article selection process.

**TABLE 3:** Key review articles exploring the connections between the built environment and healthy aging.

No.	Review article	References	Journal	Review type	Key findings
R1	“Environments for healthy aging: a critical review”	(109)	Maturitas	Critical review	The review indicates that literature has key limitations: neglect of mechanisms in person–environment relationships, lack of nationally representative samples, reliance on cross-sectional data, and poor definition of person-centered environments.
R2	Taking action on the social determinants of health in clinical practice: a framework for health professionals	(12)	Canadian Medical Association Journal	Scoping review	Proving a list of clinical practice tools that can help physicians and allied health care workers improve their performance in identifying and taking action on the root causes of poor health.
R3	Long-term exposure to residential greenspace and healthy aging: a systematic review	(66)	Current Environmental Health Reports	Systematic review	The studies suggest a potential link between long-term greenspace exposure and healthy aging but provide limited evidence. Longitudinal research on greenspace and aging indicators is needed.
R4	What is the impact of forced displacement on health? A scoping review	(103)	Health Policy and Planning	Scoping view	Forced displacement increases mortality risk, but empirical evidence on other health outcomes is often biased, limiting firm conclusions. Research could improve with better control groups and causal inference methods.
R5	The influence of the built environment on the quality of life of urban older adults aging in place: a scoping review	(113)	Built Environment	Scoping review	The findings reveal a lack of consensus on variables and tools for measuring the built environment and quality of life for older adults aging in place. Future research should prioritize creating a unified instrument to guide tailored interventions for this group.
R6	Environmental influences on healthy and active aging: a systematic review	(114)	Aging & Society	Systematic review	Research on environmental influences on active aging should adopt innovative methods, involve older adults, explore broader aspects of active aging, assess environmental characteristics, study pathways from environment to health and activity, and enhance theoretical frameworks.
R7	Impact of the built environment on aging in place: a systematic overview of reviews	(45)	Buildings	Systematic review	Thoughtful urban and housing design are pivotal in creating age-friendly environments that support aging in place
R8	Built environment and elderly population health: a comprehensive literature review	(115)	Clinical Practice and Epidemiology	Systematic review	Please write more concisely: although with some methodological limitations, the evidence reviewed in this paper suggests that some built environment variables may impact on health, especially in certain specific issues.
R9	The built environment and older adults: a literature review and an applied approach to engaging older adults in built environment improvements for health	(116)	International Journal of Older People Nursing	Critical review	The research presents the our voice framework, developed by researchers at Stanford University, as a promising strategy for engaging and empowering older people as citizen scientists, as a framework to apply to gerontological nursing and improving community health.
R10	Definitions, key themes and aspects of “aging in place”: a scoping review	(53)	Aging & Societies	Scoping review	This study concludes that the concept “aging in place” is broad and can be viewed from different (i.e., five) key themes. A more thorough understanding of “aging in place” provides knowledge about the existing key themes and aspects.
R11	Neighborhood attributes and well-being among older adults in urban areas: a mixed-methods systematic review	(104)	Research on Aging	Systematic review	Key features linked to older adults’ well-being include natural areas, adequate street furniture, community sense, good transit, and local services, which may offset adverse conditions and social deprivation.
R12	The urban environment and sustainable aging: critical issues and assessment indicators	(117)	Local Environment	Critical review	The capacity of an urban environment to support aging in place is not being assessed as an integral element of a sustainable urban environment. Identifying factors that influence healthy later life will allow the inclusion of a later-life perspective in future urban sustainability planning and assessment models.
R13	The role of building design and interiors in aging actively at home	(118)	Building Research & Information	Scoping review	Pathway and corridor design, and environmental cues that convey an instrumental function of a space also facilitated active living. Ambient features such as lighting quality and meaningful sounds and aromas were important facilitators to active living among residents with dementia.
R14	Neighborhood effects for aging in place: a transdisciplinary framework toward health-promoting settings	(105)	Housing & Society	Critical review	The neighborhood social environment has more significant relationship with older adults’ psychosocial health, the findings for neighborhood-built environment are mixed.

Source: Authors’ compilation based on literature search.

This review employs PRISMA guidelines to ensure a structured screening process adapted to prioritize studies focusing on post-industrial urban settings and their aging populations. Following the identification and screening process outlined by PRISMA for Scoping Reviews (61), 14 review articles and 28 empirical articles were deemed valid for inclusion in this review. The sampling process and the number of articles (n) are summarized in the following flowchart.

**FIGURE 2:**
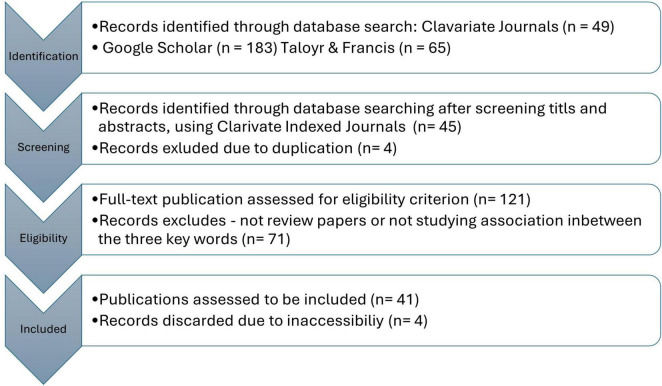
PRISMA flow diagram of the literature search on review articles and other articles as evidences.

This review has taken reference to the reporting checklist of PRISMA-ScR (Preferred Reporting Items for Systematic Reviews and Meta-Analysis Protocols) (62), which aligns with the 22 items to be reported in a typical research article or literature review. According to Figure 2, although the 42 studies examined aspects related to the urban environment’s impacts on aging, none directly concentrated on developing a paradigm of assessing aged experience specifically in the post-industrial context. The 42 studies can be divided into previous review articles and empirical studies.

Thematic clustering was guided by keywords targeting post-industrial cities, urban environments, and aging, reflecting the review’s objective to address underexplored socio-environmental interactions; three themes are hereby retrieved from the analysis: (1) existing overarching review regarding the environment and healthy aging are first presented, based on the active aging and AFCC frameworks. (2) addressing the impacts of the environment on healthy aging, using associative analysis with five sets of common indicators identified from the 42 studies to measure the process. (3) Covering the post-industrial built environment and the embedded socio-economic characteristics of post-industrial cities. The following part reports the results of these three themes mentioned above.

## 4 Analysis

### 4.1 Existing reviews on built environment and healthy aging

The existing body of literature reviews strongly focuses on at least two of the three key themes–“post-industrial cities,” “environment,” and “healthy aging.” Following the screening process against predefined eligibility criteria in Figure 2 and Table 2, 14 literature reviews are selected for analysis:

While the breadth of studies highlights the depth of research on built environment and aging, limited coverage of Global South or non-Western contexts suggests an opportunity for future research to diversify the narrative. Among the 14 selected review articles, 5 are identified as systematic or comprehensive reviews, 5 as scoping reviews, and 4 as critical reviews. Two of the review articles (R2, R9) proceed in the future by proposing a framework and guidelines for conducting further literature reviews and professional practices. This diversity in review methodologies underscores the breadth of approaches that have been applied. These reviews affirm that the built environment influences the healthy aging process through various pathways, supported by evidence of differing degrees of validity. The following section focuses on identifying the mechanisms or factors through which environmental elements impact aging, with particular attention to those most pertinent to the unique contexts of post-industrial urban environments. This will help to address the existing research gap by building a conceptual framework for analyzing aging in a post-industrial context.

### 4.2 Measuring environmental impacts on healthy aging

The review integrates environmental variables like green spaces, walkability, and air quality into a conceptual framework, linking them to theoretical constructs of healthy aging and socio-spatial dynamics. The selected articles in Theme (2) primarily examine the associations between specific environmental variables and indicators of healthy aging, rather than establishing direct causal relationships between environmental factors and aging.

Indicators of healthy aging mainly consist of variables that can be measured from an outcome perspective, such as morbidity rates, physical and mental health status, social engagement, and overall quality of life. While these studies suggest meaningful links between environmental features and aging outcomes, they do not necessarily demonstrate causation due to the complexity of endogeneity. Factors such as self-selection, reverse causality, and unobserved variables can confound this relation, making it challenging to infer direct cause-and-effect mechanisms.

Moreover, the selected articles predominantly utilize empirical data from specific geographical locations and contexts, reflecting the diversity of urban environments and worldwide demographic patterns. These contexts range from densely populated metropolitan regions to smaller, rural, or post-industrial settings, implying distinctive dynamics of socio-environmental interactions. The analysis synthesizes geographic variations, illustrating shared dynamics like the impact of green spaces while revealing contrasts in socio-environmental challenges across post-industrial regions.

The full list of geographical areas and corresponding articles included in this review is summarized in Table 4.

**TABLE 4:** Features of selected articles as evidence on association between environmental factors and health aging factors.

No.	Article	References	Environmental factors	Health aging factors	Study sites
1	Does walking explain associations between access to greenspace and lower mortality?	(71)	Walking, access to greenspace	Morbidity, life expectancy	England
2	Greenness-air pollution-physical activity-hypertension association among middle-aged and older adults: evidence from urban and rural China	(79)	Greenness, air pollution (PM_2.5_), physical activity	Hypertension	Eight selected provinces/municipalities in China
3	Heritage, health and place: the legacies of local community-based heritage conservation on social wellbeing	(119)	Community-based heritage conservation	Social well-beings	Southeast England
4	Older people, the natural environment and common mental disorders: cross-sectional results from the cognitive function and aging study	(50)	Green space and private gardens	Cognitive functions, mental disorders	UK
5	Therapeutic landscapes and wellbeing in later life: impacts of blue and green spaces for older adults	(82)	Green and blue spaces (parks, gardens, street greenery, lakes, and the ocean)	Physical, mental and social wellbeing indicators constructed through interviews	Vancouver, Canada
6	Greenness-air pollution-physical activity-hypertension association among middle-aged and older adults: evidence from urban and rural China	(79)	Residential greenness	Elderly general health	China
7	Amenity value in post-industrial Chinese cities: the case of Nanjing	(95)	Amenity value, job accessibility	Hedonic price model	Nanjing, China
8	Health benefits of pedestrian and cyclist commuting: evidence from the Scottish longitudinal study	(83)	Cycling, walkability	Hospitalization, death and prescription	Scotland (2001–2018)
9	Spatially heterogeneous associations between the built environment and objective health outcomes in Japanese cities	(120)	Mixed land-use diversity, distance to hospital, proportion of park area, and house price	Ratio of morbidity	Six Japanese cities
10	“Greening our backyard”- health behavior impacts of the built environment within the overall ecology of active living	(108)	Environmental change	Lifestyle choices	Jerusalem Railway Park
11	Bringing brain health home: the importance of housing and the urban environment for building this generation’s brain health	(121)	Housing condition	Brain health	World
12	An analysis of the connection between built environment, physical activity and health: comparing three urban neighborhoods from Shiraz, Iran	(96)	Physical form of built environment	Physical activities, obesity	Three neighborhoods from Shiraz, Iran
13	Occupational perspective of health among persons aging in the context of migration	(99)	Occupation, sense of belonging	Self-reported health	Sweden (informants migrated from Finland)
14	Social integration among adults aging with spinal cord injury: the role of features in the built and natural environment	(122)	Community built environment for supporting social integration	Spinal cord injury (SCI) condition	Midwestern United States
15	The association between perceived social and physical environment and mental health among older adults: mediating effects of loneliness	(51)	Social participation, physical environment features	Loneliness, mental health	Three cities in Flanders, Belgium
16	Contribution of the built environment to inequity in loneliness by neighborhood disadvantage in Australia	(84)	Residential density, street connectivity, and land use mix	Walkability, loneliness	Brisbane, Australia
17	Associations of neighborhood attributes with depression in mid-age and older adults: the moderating role of traffic-related air pollution and neighborhood socioeconomic status	(80)	Traffic-related air pollution (TRAP) (NO2)	Depression	Australia
18	Aging and the built environment: is mobility constrained for institutionalized older adults?	(97)	Services and amenities, location and the surrounding	Mobility	Portugal
19	Identifying built environment factors influencing the community participation of adults aging with long-term physical disabilities: a qualitative study	(81)	Built environment	Long-term physical disabilities	USA
20	Age-friendly environments and psychosocial wellbeing: a study of older urban residents in Ireland	(106)	A perception-based measure of safety, access to services, and walkability	Quality of life and eudaimonic (control, autonomy, self-realization) wellbeing (depressive mood); and social (loneliness)	Dublin, Cork, Limerick, and Galway in Republic of Ireland
21	Longitudinal associations between mental health and social environment in older adults: a multilevel growth modeling	(98)	Emotional social support, social integration, social contribution; social network and social engagement	Depression, anxiety	USA (longitudinal survey of midlife development)
22	Health disparities in Europe’s aging population: the role of social network	(112)	Social network, socioeconomic position	Self-rated health (SRH)	16 EU countries
23	Age-friendly environment, social support, sense of community, and loneliness among middle-aged and older adults in Korea	(85)	Age-friendly environment, social support, sense of community	Loneliness	Korea
24	The association of social support, depression, and loneliness with health-related quality of life in over 50 years adults: Ardakan cohort study on aging (ACSA)	(86)	Social support	Health-related quality of life (HRQoL, SF-16), duke social support index (DSSI), and center for epidemiologic studies depression scale (CES-D10)	Ardakan, Iran
25	A contemporary insight into an age-friendly environment contributing to the social network, active aging and quality of life of community resident seniors in Japan	(111)	20 factors from WHO’s active aging framework	Social network diversity, active aging, and quality of life	Japan
26	Social support, social strain and declines in verbal memory: sex-specific associations based on 16-years follow-up of the English longitudinal study of aging cohort	(123)	Quality of social connection, relation by types	Memory baseline, memory decline	England
27	Is the association between social network types, depressive symptoms and life satisfaction mediated by the perceived availability of social support? A cross-sectional analysis using the Canadian longitudinal study on aging	(124)	Types of perceived social support	Social network types, depressive symptoms and life satisfaction	Canada
28	A preliminary study of the relationship between built environment of open space and cognitive health of older people, urban regeneration strategies for enhancing livability: a case study of the Chaktai commercial area, Chattogram, Bangladesh.	(125)	Green ratio, green area size, a width of the pathway, maintenance of the whole garden, the color of green space, diversity of plants, location, and font of signage, artificial light of sitting area	Memory, concentration, judgment	Bangladesh

Source: Arranged by authors.

In Table 4, all the listed empirical articles have addressed certain aspects of the environment’s association with healthy aging. This focus on empirical evidence underlines the importance of spatial and contextual specificity in understanding the complex interplay between environmental factors and aging outcomes while also highlighting the limitations in generalizing findings and conclusions across other geographical settings.

Moreover, the literature highlights that to conduct quantitative analyses of these associations, a series of indices are employed in those empirical articles as evidence. These indices are designed to evaluate both the environment itself and the impacts of the urban environment on healthy aging, addressing various aspects of the natural and built environment, such as greenery and heritage. Many of the articles listed in Table 4 rely on measurement methods to quantify environmental features and assess their implications for health outcomes. Five of the most prominent indicators are examined in greater detail as follows:

#### 4.2.1 Environmental quality index (EQI)

Environmental quality index is an indicator adopted by the United States Environmental Protection Agency that presents data in five domains: air, water, land, built, and sociodemographic, with the county being its smallest unit of statistical representation (63). The indicator can be used to portray the overall well-being of specific places, with comparison to other geographical locations, and is capable of capturing micro-level infrastructure provisions such as local emergency department service quality or urban public park quality, which can be adapted to project the health impacts of the built environment on the elderly population (48, 64). Moreover, the index is released for public access with editions eligible for geospatial analysis; data are updated every 7 years, making it a popular data source in the listed articles.

#### 4.2.2 Urban green space indicators (UGSI)

The Urban Green Space Indicators created by WHO aim to establish a clear mechanism of pathways in linking evidence of health improvement and green spaces in urban areas – broadly defined as “natural surface” or “nature settings” such as public city parks and roadside verges (65). Nine major pathways are identified, ranging from “improved mental health and cognitive function” to “reduced cardiovascular morbidity.” The greenery’s buffering effects of noise and air pollution are particularly highlighted in the context of heavy industrial cities, reducing the risk of allergies and asthma (66). Characters of urban green spaces are further associated with specific health benefits to groups of particular demographic features by the indicators (2016, p. 18). Empirical studies have implied a more obvious connection of urban green spaces impacts on elderly groups with an average of 65+ compared to younger groups (67, 68). Evidence also indicates the strongest benefits of urban green space among the lowest socio-economic groups, which constitutes a more significant proportion of the population in post-industrial settings (69–71). The indicators address five dimensions: classification of urban green spaces, identifying measurable characteristics of urban green spaces, availability, accessibility, and usages of urban green spaces (65). It also emphasizes perception-based measures when analyzing the availability and accessibility of urban green spaces to specific demographic groups (2016, p. 27). The indicators require a rather comprehensive data set from land use to demographics and thus have only been applied in limited geographical areas such as EU countries where longitudinal data are available to construct this tool kit.

#### 4.2.3 Normalized difference vegetation index (NDVI)

The NDVI is a graphic-based indicator that measures the difference between near-infrared and red light reflectance in vegetation on the range of scale of −1.0 to +1.0 (72). It differentiates the health, density, and change of vegetation coverage into classifications of Barren (NDVI value < 0.1), Sparse (0.2–0.5), and Dense (0.6–0.9), whilst negative values indicate water, cloud, snow, or manmade structures. NDVI values are captured using remote sensing on a large geographical scale, such as the global scale, and it serves as evidence for assessing the health impacts of greenery represented by vegetation ground coverage (73), referred to as “greenery” and “greenness” in the literature in this theme.

#### 4.2.4 The index of multiple deprivation (IMD)

The IMD datasets are UK-wide small-area measures of relative deprivation across each of the constituent nations of England, Wales, Scotland, and North Ireland, published by the Consumer Data Research Center (CDRC). Although different constituent nations have applied nuances variables in data collections, the statistics are commensurable after adjustment with key indicators, including mortality rates that are highly relevant to imply a healthier aging process (74). As the deprived areas often overlap with neighborhoods within former industrial cities such as Liverpool, Manchester, Leeds, and Glasgow, it provides great insights into the aging process in post-industrial communities across the UK. It also provides a feasible set of indicators to infer the quality of life in specific areas while bestowing an overall landscape of the well-being of various vulnerable groups within particularly urban contexts. Similar data sets at the city or regional scale can be retrieved from other countries as well.

#### 4.2.5 Royal society of arts (RSA)’s heritage index

The RSA Heritage Index is a tool designed to support data-led decision-making at the local level in the UK (75). It has incorporated studies amid COVID-19 and assessed the health impacts of heritage on the elderly, particularly regarding access to heritage at a local scale (76). It implied that heritage in an “inclusive growth ecosystem” (2020, p. 9) could benefit economic development and well-being in the long term. Post-industrial cities have spaces that can be categorized as industrial heritage (77).

While these five indicators are helpful in understanding the healthy aging process in the literature, they are mainly context-specific and heavily reliant on data from Western cities, potentially overlooking environmental features in other regions. Table 4 shows a notable lack of evidence from Latin America, Sub-Saharan Africa, and Central Asia, and limited data from Eastern Europe, which might result from these region’s accessibility to English language journal publications Additionally, most studies focus on developed areas where aging poses more significant challenges to economic development. Thus, applying these findings to other contexts requires thorough examination and adaptation.

### 4.3 Challenges of healthy ageing in post-industrial cities

A synthesized thematic analysis based on the 42 literatures has shown that aging in a post-industrial context is particularly challenging, owing to the environmental characteristics embedded in the spatial-socio realities imposed by de-industrialization. The aging process in post-industrial cities in less well-off areas (e.g., South Italy) is potentially more challenging, as “socio-economic development in those areas do not keep pace with the rapid speed of population aging” [(21), p. 11]. Rooted on the unique features of the built environment and social dynamics, the positive potentials of aging in post-industrial cities are manifested in both spatial and non-spatial dimensions (78). Varies of ubiquitous challenges have been identified in the existing studies. This theme of literature shows that, apart from having different degrees of severity, socio-economic issues are often entangled with environmental problems. The following issues are particularly prevalent in post-industrial cities, significantly impacting health and aging.

#### 4.3.1 Pollution and environmental degradation

The environmental issues could be particularly challenging to the aging process in post-industrial cities due to three-fold reasons. Firstly, the former industries are often heavily polluting in nature, causing prevalent chronological diseases (13); secondly, the reduced infrastructure undermines the effective schemes in alleviating the pollution at a local level; NIMBYism means ignorance by the nearby cities that are usually in competition with the post-industrial cities for economic success; thirdly, the long-lasting nature of the environmental damages might generate other socio-economic issues such as civil unrest and distrust of the local administration. Therefore, the pollution and environmental degradation are intrinsic and multifaceted, and there is no one-size-fit-for-all solution.

From the empirical articles reviewed, air pollution in urban environments has a significant association with chronological diseases (79, 80) and long-term physical disabilities (81). Although urban greenery may have mitigating factors to self-reported health (50, 71, 82), the positive association with chronological disease and morbidity is not apparent. Furthermore, heavier air and water pollution may reduce outdoor physical activity times such as walking or cycling (83), limiting exposure to green space (66) while increasing loneliness at home (84–86), which appears to be counterproductive to the health aging process.

#### 4.3.2 Population decline and out-migration

Evidence has revealed that out-migration happens in deindustrialized societies on national (87), city (36), and neighborhood (55) scales. One of the major reasons for labor emigration from the post-industrial cities is skill mismatch, with the quick decline of labor-intensive manufacturing industrial sectors. At a city scale, youth and skilled labor emigration causes “brain drain” [(88), p. 133], leading to a vicious circle of economic consequences, such as reduced local economic activities, closures of businesses – the death of high street in the UK (89), high unemployment rate (90), the downgrading service provision (48) etc., which further contribute to socio-spatial problems of youth gang crimes (91), aggravated environmental pollution (92), exacerbated wellbeing issues both mentally and physically (46) and so on.

The impact of economic stagnation and socio-spatial deterioration as a result of de-industrialization have unequal impacts on various groups, evidences demonstrate that socio-economically disadvantaged elderly are more negatively impacted due to their limited mobility, and they are more likely to be left behind rather than voluntarily stay in, to be displaced more than autonomously moving on (42, 56, 93), leaving fewer life options with a more vulnerable aging experience. As a result, the out-migration of young people also indicates an expected decline in the birth rate in the foreseeable future (94); with the death rate among the aged population increasing, the trend of population shrinkage in these post-industrial communities may be irreversible.

#### 4.3.3 Dilapidation of urban infrastructure

The post-industrial cities’ recession is against the backdrop of neoliberalism economic policies since 2007 initiated by the institution established by the Anglo-American world (42). Under the slogan of “parsimony over prodigality,” authorities in national states, regions and cities embrace the austerity economic policy; thus, cutting down expenditures in running the city infrastructure becomes a ubiquitous practice. This policy change has profound impacts on the elderly’s lives as evidence has shown that the number and quality of amenities in the forms of urban infrastructure have positive associations with healthy aging experiences under different geographical contexts (95–97). Another strand of austerity policy is to weaken union’s power by transforming the economy from an industrial-based economy to a service-based economy; this process may lead to diminishing voices from the elderly communities of the former industrial workers, reducing their social participation (51), social and emotional support (98), and place attachment (99) thus having negative impacts on their health conditions.

One of the direct impacts of lacking financial investment in public infrastructure is the reduced quality and shrinkage of essential service provision to other “central places” (97, 100), making this infrastructure that is specifically important to the aged population no longer accessible.

Moreover, the neoliberal policy may have caused an economic downturn in a longer period (42). The stigmatization of post-industrial cities, partly caused by the dilapidated cityscape, may further prevent a prospective investor from dipping their toes (101), combined with the overarching, discouraging social factors, making it harder for the local authorities to sustain the infrastructure given a cut budget from the state government with the austerity social policies.

#### 4.3.4 Crime, exposure to Violence, and stigmatization

Aging in post-industrial communities, particularly those “undesirable neighborhoods,” [(42), p. 28] suffered from place-based stigmatizations (7). Crimes are perpetuated by the decline of the local economy and rising employment rates; laid-off former employees and youth from these families are identified as particularly vulnerable to engaging in gang culture and crimes by Fraser (91).

Another point on stigmatization is the higher percentage of the population in post-industrial areas claiming state pension, some of them perceived as physically capable of working, where a discourse of immorality is pervasive (102). Those areas, as a whole, often received more state bursaries supported by taxpayers nationwide, which may further provoke dissatisfaction and social pressure from other economically prosperous cities and regions (53). Nevertheless, the elderly may not receive much helpful financial aid due to the potential enormous financial burden and an unpredictable return of invest by the state administration and local government, given the aggravated economic shock, underlying health problems and a lack of social support (103).

The unemployment-as-a-lifestyle narrative constructs an image of an unethical group that is taking advantage of the state welfare system deliberately; it can be further combined with the crime-and-violence discourse in the post-industrial neighborhood (104, 105), strengthening the reputation of people and communities in post-industrial societies as lazy, dangerous, and undesirable, thus reducing the social support locally and from the regionals in affinity (86, 103).

The violence in post-industrial settings can be regarded as antagonism as well as self-protection and may cause accidental mortality or morbidity among vulnerable elderly groups. Furthermore, it reduces the perceived safety, which is highly associated with access to services and walkability, particularly at night time (106). The knocking-on effect of street violence and crimes preventing the elderly from exercising on their own due to the fear of falling victim to violent crime is counterproductive to maintaining their health (52).

Within a de-industrialized city, the “rough” is separate from the “respectable” (107), creating another layer of spatial exclusion. Moreover, local authorities’ inappropriate policy intervention could further alienate the groups considered odd and violent, either by “dumping” them into more deprived areas or mixing them with divergent groups. This fails to address the essential issues that result in the problems (103, 108). As a result, the overall safety of the areas and the cities could further deteriorate.

## 5 Conclusion

The previous section explored themes addressing the mechanisms and dimensions influencing the healthy aging process in post-industrial cities, emphasizing the importance of understanding the interplay between the built and socio-economic environments. The following sub-sections evaluate the credibility and limitations of this review while proposing directions for unlocking the potential of post-industrial cities to support healthy aging.

### 5.1 Evaluation of the evidence

**FIGURE 3:**
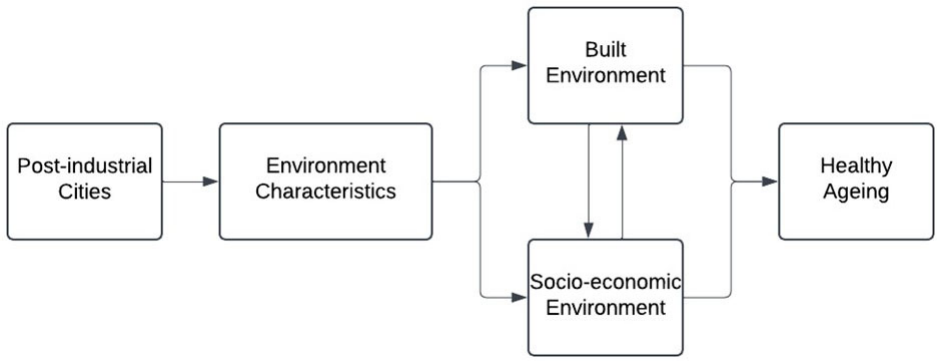
A framework of studying healthy ageing in post-industrial cities.

The above analysis delves into three key areas regarding the research question: an overview of the built environment’s impacts on healthy aging, measuring these impacts by studying associations with existing environmental and health variables and examining the socio-environmental features of post-industrial cities. Based on the three themes identified in the analysis, a framework for researching healthy aging in post-industrial cities is proposed, as shown in Figure 3.

Post-industrial cities influence healthy aging through interrelated environmental and social characteristics, such as green spaces and strong social networks, which are synthesized here to reveal untapped opportunities for improving elderly well-being. The environment includes both the built and socio-economic environments, which influence each other and have both short-term and long-term effects on healthy aging. Studies have shown associations between various environmental factors, such as greenery (built environment), access to public spaces (built environment), street crime (socio-economic environment), and a sense of community (socio-economic environment). This review enhances the understanding of the relation between “post-industrial cities” and “environmental characteristics” by examining factors in both the built and socio-economic environments that previous studies have overlooked. It focuses on the context of post-industrial realities, considering de-industrialization and aging, while synthesizing the impact of environmental factors on healthy aging through two interconnected pathways.

To summarize, the evidence has shown that (1) environment has a close association with healthy aging, casting lopsided impacts on the elderly than their younger counterparts; (2) post-industrial cities and communities have peculiar spatial features that induce unique socio-economic features; (3) those socio-spatial features are mostly perceived hostile toward the healthy aging process using existing environmental-health indicators.

### 5.2 Limitation of the evidence

Apart from the achievement in building an overarching analytical framework for studying the environmental impacts of post-industrial cities on healthy aging, this review has identified both conceptual and methodological limitations of the existing review literature and empirical studies. Moreover, regarding the data set assessed in the reviews, cross-sectional data are applied, while the assessment of the long-term healthy aging process will need a traceable longitudinal data set [(109), p. 16].

Furthermore, the review scope has focused on evidence published in English language, which result to the relative less discussion in geographical areas out of the Anglophone-sphere. For instance, evidence on post-industrial cities and communities produced by scholars located in Latin American is primarily written in Spanish and Portuguese, with similar circumstance for other regions identified as lack of review evidence. This data limitation calls for further review on evidence discussing post-industrial cities and healthier aging from literature in other major languages.

In Figure 3, when establishing the connection between built environment as well as socioeconomic environment with healthy aging, we have identified three caveats, which worth further elaboration when evaluating the potential credibility of our findings. Firstly, those perceived “negative aspects” of the built environment are not exclusively possessed by post-industrial cities, or in other words, post-industrial cities do not need to be “deprived and dilapidated,” and there are counter facts from across the globe (i.e., post-industrial but having good environment) (77, 110). Secondly, social factors are usually the original reason for preventing access to environmental assets of vulnerable groups. Still, the causality of a dilapidated built environment leading to social problems is hard to prove (101) – due to the nature of social issues such as violence or gang culture, which are often interwoven. Thirdly, the interpretation of the impacts based on data or evidence could be biased – the impacts are usually reported by left-behind groups or researchers about other non-industrial cities, which are in the tread of economic growth. It could be unfair to compare cities on the rise, such as those financial hubs, and cities in decline, such as post-industrial cities. These three caveats may requires further evidences to come to more accurate understanding of the potential interactions of built and socio-economic environments’ impacts on healthy aging under post-industrial reality.

As a result, we argue that although socio-spatial factors may have negative health impacts on the aged population, post-industrial cities have great potential to generate positive health impacts on the aging process and results. These can be identified from synthesizing existing evidence, which can be further articulated from both spatial and social aspects.

### 5.3 Potentials of post-industrial cities for healthy aging

#### 5.3.1 Spatial potentials

The reviewed articles have consistently demonstrated a strong and reliable link between access to and use of heritage and public spaces and improvements in the health and well-being of elderly populations. This connection is particularly evident through engagement in recreational activities and physical exercise, which are often facilitated by these spaces. By participating in such activities, older adults benefit from the physical exercise and the social interaction, mental stimulation, and overall sense of community provided by such spaces. Therefore, heritage and public spaces play a crucial role in enhancing the quality of life and promoting healthy aging, particularly for those facing mobility challenges or other health issues.

Post-industrial cities, with their legacies of former industrial sites, offer significant potential for space transformation into cultural and recreational infrastructure, such as museums, theme parks, or open public spaces, provided they undergo thoughtful planning, design, and regeneration. Studies have shown that repurposing these sites can effectively capitalize on the existing spatial structures and infrastructure, including elements like drainage systems, reducing overall regeneration costs. Additionally, former industrial “brownfield” sites–often left underused after the relocation of factories–can be converted into green spaces or vibrant social public spaces to leverage their health benefits, as shown in theme (2) of this review. Both heritage and green spaces have been linked to significant benefits in promoting healthy aging, providing environments that encourage physical activity, social interaction, and mental well-being. The revitalization of these spaces not only offers a sustainable use of land but also represents an opportunity to improve the health outcomes of an aging population in a post-industrial setting, with existing examples such as the National Mining Museum in Edinburgh, Scotland, and Shizuoka in Japan.

#### 5.3.2 Social potentials

Despite being perceived as unproductive and inactive in economic activities, the elderly can serve as a potential reservoir of labor for the market if their previous skills and experience are transferred through proper outlets (78). The nature of work does not necessarily need to be salary-based; it can include community work, freelancing, or voluntary services. A good example is, former miners could serve as cultural tour guides at local industrial heritage at Scotland National Museum of Mining in Edinburgh.

In addition to the relationships nurtured by geographical proximity and time for the aging population, post-industrial cities are home to communities with shared occupational identities, such as “co-workers,” or even a sense of “working-class identity” united by unions. These special social networks, which intertwine factory work and family life in the neighborhood, have preserved a collective memory and have been proven to be beneficial in maintaining good mental and physical well-being during the aging process. These established social ties and networks of support have significantly reduced loneliness and the risk of depression (51).

Last but not least, the tightly maintained social networks among the elderly in post-industrial cities can be leveraged to foster greater civil participation (111, 112). The elderly can also engage in local politics and urban governance, assuming roles as local elected officials, which aligns with the WHO’s framework on Active Aging (77).

### 5.4 Recommendations

The findings of this critical literature review call for further studies on three aspects. Firstly, there is a need to clarify the scale and extent of the interactions between the built environment and the social conditions in post-industrial cities (42). Secondly, the dynamics should be examined to establish clearer causality between environmental and health variables and pathways and mechanisms by which the built environment’s social consequences impact health. Thirdly, it is important to explore how unique post-industrial cities are as platforms for influencing the healthy aging process. All these areas of further research can potentially expand the scope and depth of contemporary conceptualizations, theories, and research frameworks concerning healthier aging processes. Additionally, longitudinal data may need to be collected with collaborations from local authorities, NGOs and health organization and compare globally across various types of post-industrial cities and communities to triangulate the existing evidence. Researchers from higher educational institution and research center can play a more active role in engaging the stakeholders and explore the generatable findings based on local dataset. Community engagement at a local level is also essential to wide participation of such survey and implement potential changes that help to improve the aging experience of the elderly residents.
