# Correction: Urban environments' impacts on aging in post-industrial cities: reviewing the pathways to healthy aging

**DOI:** 10.3389/fmed.2025.1760229

**Published:** 2026-01-05

**Authors:** Hongsheng Zhao, Marco Spada, Ilaria Bortone

**Affiliations:** 1 School of Technology, Business, and Arts, University of Suffolk, Ipswich, United Kingdom; 2 School of Computing, Engineering and Physical Sciences, University of the West of Scotland, Paisley, United Kingdom; 3 APS Public Health Environment and Social Equity - PLANET, Bari, Italy

**Keywords:** healthy ageing, urban environment, age-friendly cities, and communities, active ageing, post-industrialization

Affiliation School of Computing, Engineering and Physical Sciences, University of the West of Scotland, Paisley, United Kingdom was erroneously given as School of Computing, Engineering and Physical Sceinces, University of the West of Scotland, Paisley, United Kingdom.

An incorrect **Funding** statement was provided. The correct funding statement reads:

The author(s) declared that financial support was received for this work and/or its publication. This work was supported by the MISTRAL Project (Horizon Europe, Grant Agreement No. 101095119) and by the TCH—Taranto Community Hub Project (CUP B95I23000430009). The Article Processing Charge was funded by the TCH—Taranto Community Hub Project (CUP B95I23000430009).

The original version of this article has been updated.

